# Transdiagnostic Biomarkers and Mechanistic Heterogeneity in Cognitive Fatigue: A Response to Rudroff

**DOI:** 10.1017/S0033291726104929

**Published:** 2026-06-18

**Authors:** Stefanie Linnhoff, Roi Cohen Kadosh, Tino Zaehle

**Affiliations:** 1 Institute for Medical Psychology, Otto-von-Guericke University, Magdeburg, Magdeburg, Germany; 2 Department of Neurology, Otto-von-Guericke University, Magdeburg, Germany; 3 School of Psychology, University of Surrey, Guildford, UK

Dear Editor,

In our recent article, we reported that the frontal aperiodic exponent of the EEG power spectrum may serve as a transdiagnostic biomarker of cognitive fatigue across multiple sclerosis and Long COVID (Linnhoff et al., 2026). Dr. Rudroff subsequently provided a thoughtful commentary on our findings, raising important considerations regarding population diversity and neurobiological heterogeneity in biomarker research (Rudroff, 2026). We thank Dr. Rudroff for his thoughtful and conceptually rich commentary and for engaging so constructively with our work. We appreciate the opportunity to further contextualize our findings and agree that the issues raised, particularly regarding population diversity and neurobiological heterogeneity, represent important considerations for the development of clinically meaningful biomarkers. At the same time, we would like to clarify how these points relate to the scope and interpretation of our study.

We fully agree that broader demographic representation is an important goal for future research. Our study was conducted in a relatively homogeneous, single-center sample, and we acknowledge that this limits the extent to which our findings can be directly generalized to more diverse populations. Importantly, however, our intention was not to propose a universally generalizable biomarker, but rather to provide initial evidence for a reproducible electrophysiological correlate of fatigue within a well-characterized sample. In line with this, we framed the aperiodic exponent as a promising, but not yet definitive, marker and emphasized that it should be considered as complementary to existing approaches rather than as a standalone diagnostic tool. We, therefore, fully concur that future studies should explicitly examine whether this marker performs consistently across more diverse demographic and clinical populations.

We also agree that fatigue is unlikely to be mechanistically uniform. Indeed, one of the central challenges in fatigue research is that similar subjective symptom profiles may emerge from distinct biological pathways, and the PET findings cited by Dr. Rudroff are therefore highly relevant in highlighting this heterogeneity (Rudroff, 2024). At the same time, as with our own work, these findings will require replication in sufficiently powered studies to establish their robustness and generalizability (Button et al., 2013). Our study was not intended to demonstrate mechanistic equivalence between multiple sclerosis and Long COVID, nor to suggest that a single electrophysiological marker fully captures all causal pathways leading to fatigue. Rather, we aimed to identify a robust electrophysiological correlate of subjective cognitive fatigue at the systems level. As outlined in our manuscript, the mechanistic interpretation of the aperiodic exponent remains indirect and should be considered with caution.

From this perspective, similar EEG signatures may arise from different underlying biological mechanisms without undermining their relevance as biomarkers. Instead, the aperiodic exponent may reflect a convergent functional expression of fatigue across distinct etiologies. Accordingly, a shared electrophysiological pattern across multiple sclerosis and Long COVID does not imply a single pathophysiological mechanism, but rather partially overlapping alterations at the level of cortical dynamics. More broadly, biomarkers do not necessarily need to map one-to-one onto a single underlying mechanism to be clinically useful. In many areas of medicine, they are valued because they reliably index a clinically relevant state, even when the biological pathways leading to that state are heterogeneous.

Within this framework, apparent differences between EEG- and PET-derived findings can be understood as reflecting distinct levels of neural organization rather than contradictory results. More generally, different neuroimaging modalities provide distinct and only partially overlapping windows into neural functioning, and direct comparisons between them should therefore be made with caution (Cohen Kadosh, 2025). While PET may reveal heterogeneity in metabolic or neurochemical processes, the aperiodic exponent may capture shared alterations in cortical population dynamics or excitation–inhibition balance at the electrophysiological level. Thus, divergence at one level of measurement does not preclude convergence at another. Instead of representing competing explanations, these approaches provide complementary perspectives on brain function, and their integration will likely be essential for a more complete understanding of fatigue. In this context, an important next step will be to examine how such markers behave under intervention. Assessing whether electrophysiological and metabolic measures show convergent or divergent changes in response to targeted treatments may provide a more direct test of their functional and clinical relevance.

We also appreciate the point that average classification metrics may obscure subgroup variability. Our reported AUC reflects aggregate discriminative performance and does not imply equal sensitivity across all biological or demographic subtypes. In the manuscript, we therefore interpreted the observed classification performance with caution and highlighted its current limitations, including the moderate sensitivity and specificity and the need for further validation. Future studies that examine subgroup-specific performance, particularly in combination with multimodal approaches, will be important to better understand this variability.

Overall, we appreciate the points raised in this commentary as they help to situate our findings within a broader conceptual framework. Our study was intended as a first step toward identifying an objective, clinically accessible electrophysiological correlate of fatigue across conditions, while explicitly acknowledging its current limitations and the need for further validation. We therefore view the considerations of population diversity and neurobiological heterogeneity not as contradictions of our findings, but as important directions for future research that build on the present work.

Sincerely,

Stefanie Linnhoff, Roi Cohen Kadosh, and Tino Zaehle
